# Band Gap Narrowing by Suppressed Lone-Pair Activity of Bi^3+^

**DOI:** 10.1021/jacs.4c00150

**Published:** 2024-02-23

**Authors:** Kanta Ogawa, Ryu Abe, Aron Walsh

**Affiliations:** † Department of Materials, Imperial College London, Exhibition Road, London SW7 2AZ, U.K.; ‡ Department of Energy and Hydrocarbon Chemistry, Graduate School of Engineering, Kyoto University, Nishikyo-ku, Kyoto 615-8510, Japan

## Abstract

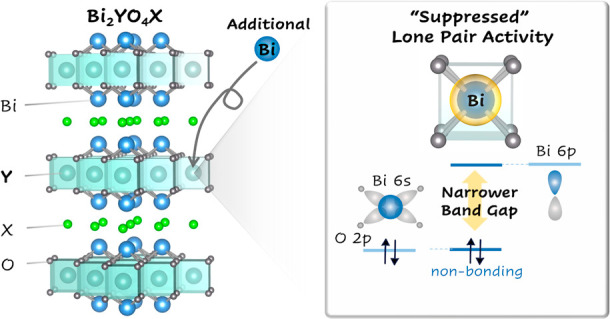

Post-transition metal cations with a lone pair (*n*s^2^*n*p^0^) electronic configuration such as Pb^2+^ and Bi^3+^ are important components of materials for solar-to-energy conversion. As in molecules like NH_3_, the lone pair is often stereochemically active in crystals, associated with distorted coordination environments of these cations. In the present study, we demonstrate that suppressed lone pair stereochemical activity can be used as a tool to enhance visible light absorption. Based on an orbital interaction model, we predict that a centrosymmetric environment of the cations limits the orbital interactions with anions, deactivates the lone pair, and narrows the band gap. A high-symmetry Bi^3+^ site is realized by isovalent substitutions with Y^3+^ by considering its similar ionic radius and absence of a lone pair. The quaternary photocatalyst Bi_2_YO_4_X is singled out as a candidate for Bi substitution from a survey of the coordination environments in Y–O compounds. The introduction of Bi^3+^ to the undistorted Y^3+^ site in Bi_2_YO_4_X results in a narrowed band gap, as predicted theoretically and confirmed experimentally. The orbital interaction controlled by site symmetry engineering offers a pathway for the further development of post-transition metal compounds for optoelectronic applications.

Semiconductors containing post-transition metal cations with a lone pair (*n*s^2^*n*p^0^) electronic configuration (Sn^2+^, Sb^3+^, Pb^2+^, Bi^3+^) are a special class of photoabsorbers in solar-to-energy conversion systems.^1−4^ The filled s orbitals interact with anion orbitals to form the upper valence band, providing unique optoelectronic properties such as defect tolerance,^5^ reduced hole effective mass,^6,7^ and shallow ionization potentials. These effects have led to promising photo(electro)catalysts such as BiVO_4_,^8,9^ Bi_4_NbO_8_Cl,^10,11^ and Pb_2_Ti_2_O_5.4_F_1.2_.^12^

The orbital interactions in these materials can be described within the revised lone-pair model,^1^ which explains the asymmetric coordination environment of these cations in many crystal structures.^13−18^ The metal s–anion p interactions create filled bonding and antibonding combinations as illustrated in Figure 1. The antibonding combination further interacts with the empty metal p orbitals.

**Figure 1:**
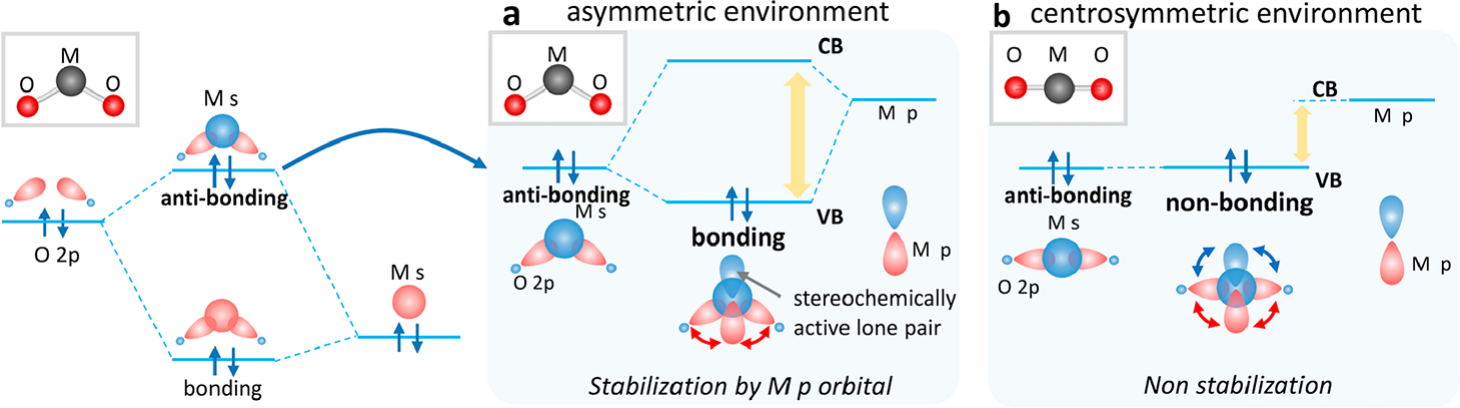
Illustration of the band-edge orbital interactions in lone pair containing metal oxides. The metal (M) s–anion (O) p interactions create filled bonding and antibonding combinations. (a) When the coordination environment of the cation is asymmetric, the antibonding combination is stabilized by M p coupling to form the upper valence band (VB). This results in a stereochemically active lone pair due to the M p orbital contribution to the upper VB. (b) When the coordination environment of the cation is centrosymmetric, this orbital interaction is suppressed due to destructive interference, which results in a narrower band gap. In this case, the antibonding combination between M s–O 2p mainly forms the upper VB without M p orbital contribution.

The extent of the metal p orbital mixing is influenced by the coordination environment of the cation. At an asymmetric site, the mixing is favorable as shown in Figure 1a, providing the stereochemically active lone pair. The distorted cation coordination environments stabilize the valence band maximum (VBM) and enlarge band gap as seen in δ-Bi_2_O_3_^19,20^ and SnWO_4_.^21^ Conversely, we posit that when a lone pair cation is placed at a high symmetry site, the band gap will be narrowed due to suppressed metal p coupling (Figure 1b). One approach to achieve a higher symmetry environment is through external force. Balic-Zunic et al. studied the effect of pressure on the crystal structure of Bi_2_S_3_, showing that high pressure provides a more symmetric coordination environment of Bi^3+^.^22^ A subsequent theoretical study by Olsen et al. revealed that the symmetry increase is accompanied by a reduced band gap of Bi_2_S_3_.^23^ However, high-pressure is impractical for solar-to-chemical conversion systems. Here, we demonstrate that a high symmetry Bi^3+^ can be realized under ambient conditions, which results in a significant reduction of the band gap.

We focus on yttrium because Y^3+^ has a similar ionic radius (1.02 Å) to Bi^3+^ (1.17 Å)^24^ but without the valence s^2^ electrons causing the structural distortion. From the Materials Project database,^25^ we searched for metal oxide crystals with Y^3+^ in a highly symmetric site and then investigated the effect of the Bi^3+^ introduction to the Y^3+^ site. We analyzed the coordination environments of Y^3+^ based on the Voronoi approach and the Continuous Symmetry Measure (CSM), where the environment is expressed as the most similar model polyhedron with the CSM value.^26^ The CSM ranges from 0 (perfect polyhedron) to 100 (highly deformed). Undistorted Y^3+^ with small CSM values were found in the 6-fold octahedral (O:6) and 8-fold cubic (C:8) environments (Figure S1).

For O:6, the double perovskites Ba_2_Y*M*O_6_ (M = Nb, Ta, Sb) provide regular environments (CSM = 0). The effect of Bi^3+^ substitution onto the Y^3+^ sites was investigated based on density functional theory (DFT) calculation within generalized gradient approximation. The band gaps are substantially reduced in each case (Figure S2) by 0.6–1.9 eV. The negligible Bi 6p but strong Bi 6s contributions to the VBM are consistent with our model (Figure 1b). The Bi^3+^-based materials in the database were also analyzed (Figure S3), where the Bi^3+^ environment in Ba_2_Bi*M*O_6_ (M = Sb, Ta) is slightly more distorted (CSM = 0.02, 0.03) than the Y^3+^ in the corresponding Y compound. The most distorted Bi^3+^ is accompanied by the strong Bi 6p contribution to the upper valence band (Figure S4) consistent with the orbital interaction model in Figure 1a. Note that a similar tendency can be observed for bivalent Pb^2+^ (Figure S5).

For C:8, the layered oxyhalides Bi_2_YO_4_X (X = Cl, Br, I; Figure 2a) show regular environments (CSM = 0).^27^ The Bi introduction exerts a similar influence on their electronic structure with a band gap reduction of 0.3–0.6 eV. Note that no crystal structure was found with the CSM close to 0 in the C:8 environment of Bi^3+^, showing that Bi^3+^ at an undistorted C:8 environment is rare (Figure S6).

**Figure 2:**
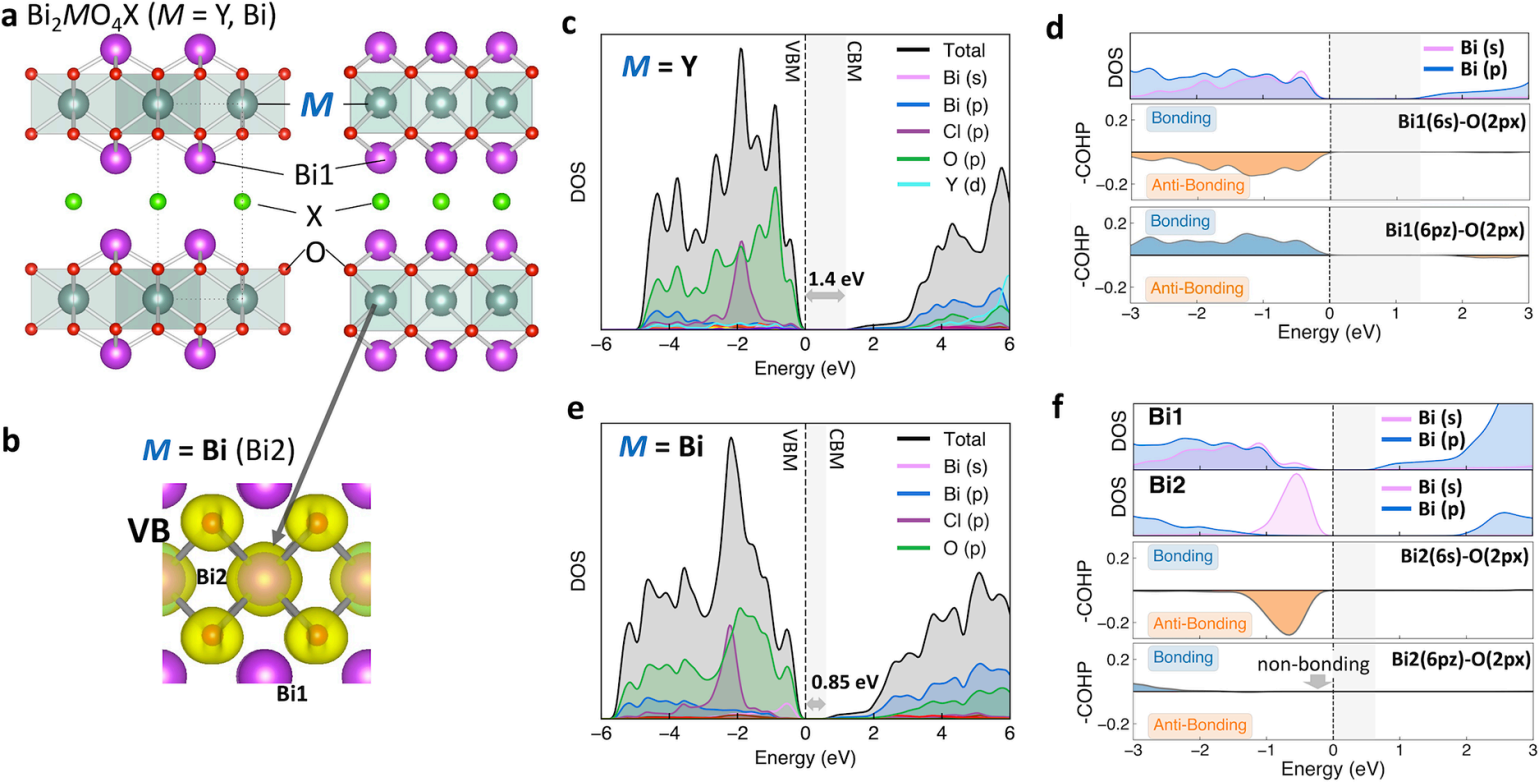
(a) Crystal structure of Bi_2_YO_4_X. (b) Charge density distribution of the upper part of the valence band (VB) for Bi_2_*M*O_4_Cl with M = Bi. The Bi at the *M* site is described as Bi2, while the Bi site in original Bi_2_YO_4_X is described as Bi1. Projected electronic density of states (DOS) of Bi_2_*M*O_4_Cl with M = (c, d) Y and (e, f) M = Bi with crystal orbital Hamilton populations (COHP) for Bi1 (Bi2) and O 2p. The highest occupied state (Fermi level) is set to 0 eV.

We selected Bi_2_YO_4_X (X = Cl, I) which features a cubic Y^3+^ site as a target for Bi^3+^ introduction. The oxychloride has been reported as a water-splitting photocatalyst.^28^ In Bi_2_YO_4_Cl, Bi1 contributes to the VBM (Figure 2c, d), in the manner expected for a stereochemically active lone pair (Figure 1a). The interactions between Bi1 and O 2p were confirmed by Crystal Orbital Hamiltonian Population (COHP) analysis (Figure 2d).^29^

The introduction of excess Bi on the Y site (i.e., forming Bi_2_BiO_4_Cl) significantly reduced the calculated band gap from 1.4 to 0.9 eV. The new VBM is derived from the 6s orbital of cubic Bi2. The s-orbital character around Bi2 is seen in the VBM electron density (Figure 2e, f). The COHP analysis shows the antibonding character between Bi2 6s and O 2p and nonbonding between Bi2 6p and O 2p (Figure 2f). The high-symmetry Bi^3+^ narrows the band gap by restricting the Bi6p participation to the valence band (Figure 1b). A similar tendency was observed for Bi_2_YO_4_I (Figure S7). It is noted that the Bi introduction to Y site negligibly affects the CBM nature because the conduction band is derived from the highly dispersive interlayer Bi1–Bi1 interaction but not from the Bi2 (Figure S8).^30^

The Bi introduction to the Y site affects other physical properties. The hole effective mass is enlarged especially in the out-of-plane direction (*c* axis) owing to the nonbonding character of the Bi2 6s orbital, while the electron effective mass is hardly affected as stated above (Table S1). The nonbonding character of Bi2 6s orbital also provides a relatively flat phonon band related to the isolated motion of Bi2; however, no imaginary modes are found (Figure S9). The Born effective mass of Bi2 is also more symmetric; the larger average value of Bi2 (4.8) than Y (4.2) is typical of high-polarizability lone pair cations. This will enhance the dielectric screening, which can be an advantage for charge carrier dynamics (Table S2).^31^

We experimentally introduced Bi to the Y site in Bi_2_YO_4_X. The Bi excess compounds, Bi_2_Bi_*x*_Y_1–*x*_O_4_X, were synthesized by solid-state reaction. Doping above *x* = 0.5 for X = Cl and *x* = 0.4 for X = I provided impurity phases such as Bi_3_O_4_Cl and Bi_4_O_5_I_2_ whose Bi site is asymmetric (Figure S10). Calculations within the quasi-harmonic approximation suggest that a dynamic instability emerges for expanded volumes at high temperatures (Figure S11). This suggests that low-temperature processing may increase the solubility of excess Bi.

Rietveld analysis of synchrotron X-ray diffraction (SXRD) patterns of the synthesized Bi_2_Bi_*x*_Y_1–*x*_O_4_X (*x* = 0, 0.2, 0.4, 0.5 for X = Cl, *x* = 0, 0.1, 0.2, 0.4 for X = I) shows the Y/Bi ratios at Y site (Bi2) consistent with *x* values, confirming the successful doping of Bi to the Y site (Figures 3a–c, S12, S13). The Bi-incorporation at the Y site was also supported by HAADF-EDX analysis with an increased Bi count at the Y site (Figure 3d,e) and SEM-EDX mapping (Figures S14, S15). Note that the cell volume was expanded with increasing *x* value because of the larger ionic radius of Bi^3+^ (Figure S16).

**Figure 3:**
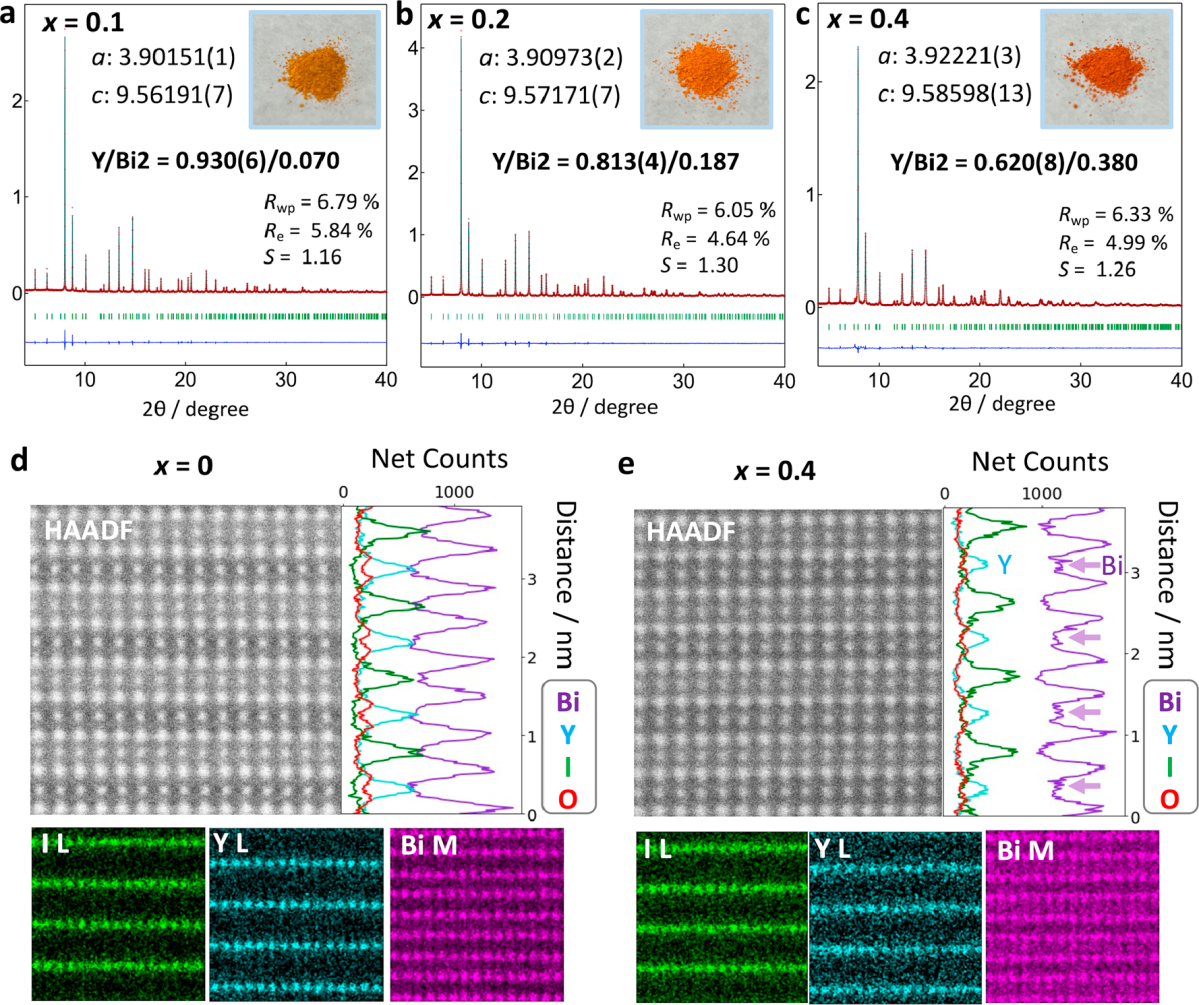
SXRD patterns and the Rietveld refinement profiles and product color of Bi_2_Bi_*x*_Y_1–*x*_O_4_I (*x* = (a) 0.1, (b) 0.2, (c) 0.4). The structure model is based on Bi_2_YO_4_I (*P*4/*mmm*).^27^ HAADF images of Bi_2_Bi_*x*_Y_1–*x*_O_4_I (*x* = (d) 0, (e) 0.4) along the [110]_t_ direction with STEM-EDX line scan analysis along the [001] direction and atomic resolution elemental maps for Y (light-blue), I (light-green), Bi (purple). Bi peaks can be observed around Y site in the doped sample (e).

The Bi introduced into the Y site significantly narrows the band gap (Figure 4), accompanied by a newly formed density of states increasing with the increased Bi ratio (Figure S17), and a change in the color of the powder (Figure 3a–c). Though a similar band gap narrowing was reported for the oxychloride system, the underlying mechanism remains elusive.^32^ The band edge positions were estimated based on the lowest ionization energy obtained by photoelectron yield spectroscopy (PYS). The VBM increases with Bi doping to the Y site (Figure 4). In Bi_2_YO_4_X (*x* = 0), the excess Bi causes a negative VBM shift, which is more significant in the oxychloride than the oxyiodide. For *x* = 0, the oxyiodide shows a narrower band gap than the chloride owing to the I 5p contribution to the VBM, while O 2p mainly contributes to the VBM of the chloride. In other words, in the oxyiodide, I 5p on the VBM of *x* = 0 mitigates the observed VBM shift by Bi introduction to Y, while, in the oxychloride, the effect of the change in Bi coordination environment from asymmetric (Bi1) to symmetric (Bi2) on the VBM shift can be seen. On the other hand, the CBM remains weakly affected because the conduction bands are derived from the interlayer Bi1–Bi1 interaction, whose highly dispersive nature overwhelms the contribution of the Bi2 6p to the CBM as shown in the density of states in Figure 2. The lower conduction bands of the iodides than the chlorides are derived from the larger Bi1–Bi1 distance, which results in the narrowed conduction bandwidth.^30,33^

**Figure 4:**
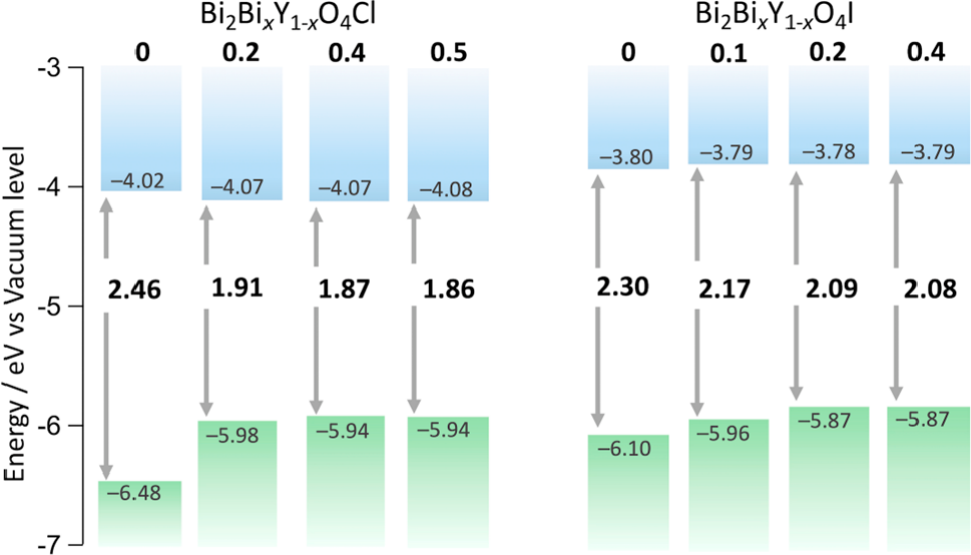
Band edge positions of Bi_2_Bi_*x*_Y_1–*x*_O_4_X (*x* = 0, 0.2, 0.4, 0.5 for X = Cl, *x* = 0, 0.1, 0.2, 0.4 for X = I). The lowest ionization energies obtained via photoelectron yield spectroscopy (PYS) are assumed to the valence band maximum.

We have demonstrated the connection between site symmetry, orbital interactions, and band gap in lone-pair-containing compounds. The predicted effects of high-symmetry Bi^3+^ sites were validated from synthetic experiments on Bi_2_YO_4_X with additional Bi substituted on the Y sites. This cation substitution approach can be extended to other combinations such as Sc/Sb, Ca/Sn, and Sr/Pb. Lone pair engineering offers a strategy for controlling the optoelectronic structure of the post-transition metal compounds beyond the limits of known materials.

## Supporting Information Available

Experimental section, additional characterization, theoretical calculation analysis (Figures S1–S17) (PDF)
