# Supplementary material for: Band Gap Narrowing by Suppressed Lone-Pair Activity of Bi3+
Source: J Am Chem Soc. 2024 Feb 23;146(9):5806–10. doi: 10.1021/jacs.4c00150 (PMC10921403; doi:10.1021/jacs.4c00150)
Supplement: Supplementary file 1 — ja4c00150_si_001.pdf [file ja4c00150_si_001.pdf]

*Supporting Information*

# Band Gap Narrowing by Suppressed Lone-pair Activity of $Bi^{3+}$

Kanta Ogawa,*[1] Ryu Abe,[2] and Aron Walsh[1]

[1] Department of Materials, Imperial College London, London SW7 2AZ, UK

[2] Department of Energy and Hydrocarbon Chemistry, Kyoto University, Kyoto 615-8510, Japan

## METHODS

### Coordination Environment Analysis

The coordination environments of $Y^{3+}$ and $Bi^{3+}$ compounds present in the Material Project database[1] were analyzed. We selected stable materials (on the convex hull) containing at least $Y^{3+}$ (or $Bi^{3+}$) with a band gap larger than 0.5 (0 for $Bi^{3+}$) eV. The coordination environment was analyzed based on the Voronoï approach and the Continuous Symmetry Measure (CSM) using Chemenv[2] in the pymatgen package,[3] where the environment is expressed as the most similar model-polyhedron with the CSM value.[2] The CSM ranges from 0 to 100, where 0 shows a perfect, i.e., undistorted, polyhedral environment.

### Material Modelling

All calculations were performed using Density Functional Theory (DFT) within periodic boundary conditions through the Vienna Ab Initio Simulation Package (VASP).[4] The projector augmented-wave (PAW) method was employed. The Perdew-Burke-Ernzerhof (PBE) formulation of the generalized gradient approximation (GGA) was employed as the exchange-correlation functional. D3 dispersion was included for layered oxyhalides, considering the Van Der Waals interaction between layers. The atomic positions were optimized until the Hellman−Feynman forces on each atom were below 0.001 $eVÅ^{-1}$. The energy convergence criterion was set to $10^{-5}$ eV. A plane-wave energy cut-off of 600 eV was used for all calculations. A 7 × 7 × 3 Γ-centred k-point mesh was employed for geometry optimization of $Bi_2MO_4X$ ($M$ = Y, Bi, X = Cl, I; $P4/mmm$) and was doubled for projected density of states (PDOS) and crystal orbital Hamilton population (COHP) calculations using the LOBSTER package. 4 × 4 × 4, 4 × 4 × 4, 4 × 4 × 3, 3 × 3 × 5, 3 × 3 × 5, 4 × 4 × 5 Γ-centred k-point meshes were employed for $Ba_2BiNbO_6$ ($Fm\bar{3}m$), $Ba_2BiSbO_6$ ($Fm\bar{3}m$), $Ba_2BiTaO_6$ ($I4/m$), $Ba_2BiSbO_6$ ($C2/m$), $Ba_2BiTaO_6$ ($C2/m$), $Al_4Bi_2O_9$ ($Pbam$) respectively. The conductivity effective mass tensors were calculated using AMSET.[5]

The dielectric constants were calculated by density functional perturbation theory (DFPT). Electronic band structure diagrams were generated using the sumo package.[6]

To calculate the vibration frequencies, a 4 × 4 × 2 supercell was built and force constants were calculated by the method of small displacements implemented in Phonopy.[7] The Helmholtz free energy calculation in the quasi-harmonic approximation was also made using the Phonopy software.[8]

## Synthesis

$Bi_2Bi_xY_{1-x}O_4X$ ($x$ = 0 – 0.5) were prepared by prepared by solid-state reactions. $Bi_2O_3$ (FUJIFILM Wako Pure Chemical Corporation, 99.99%), $Y_2O_3$ (FUJIFILM Wako Pure Chemical Corporation, 99.99%), and BiOX were mixed at the ratio of 1+$x$: 1–$x$: 2, heated in an evacuated silica tube at 1073 K (1023 K for $Bi_2YO_4I$ and $Bi_2Bi_{0.6}Y_{1-0.6}O_4I$ ) for 10h. BiOCl was purchased from FUJIFILM Wako Pure Chemical Corporation, while BiOI was synthesized by a soft liquid deposition method;[9] 5 mmol of $Bi(NO_3)_3 \cdot 5H_2O$ (FUJIFILM Wako Pure Chemical Corporation) was dispersed in 30 mL of ethanol and mixed with the solution of 5 mmol of KI (FUJIFILM Wako Pure Chemical Corporation) dissolved in 10 mL of pure water. After 5 h stirring at room temperature, the precipitate was collected by centrifugation, washed several times with water and ethanol, and finally dried in air at 333 K.

## Characterization

Powder XRD (MiniFlex II, Rigaku, X-ray source: Cu Kα), UV−visible diffuse reflectance spectroscopy (V-650, JASCO), SEM-EDX (NVision 40, Carl Zeiss-SIINT) were used for the characterization of samples. High-angle annular dark-field scanning transmission electron microscopy (HAADF-STEM) and annular bright-field scanning transmission electron microscopy (ABF-STEM) images were collected using a JEM-ARM200CF (JEOL Ltd., Tokyo, Japan) operating at an accelerating voltage of 200 kV and equipped with a cold field emission gun and a Cs corrector to observe atomic columns. Elemental analysis was carried out using JEM-ARM200CF equipped with energy-dispersive X-ray spectroscopy (EDX). Samples were prepared by grinding the material and depositing a few drops of the suspension onto a holey copper grid covered with a thin carbon film. Synchrotron X-ray diffraction (SXRD) patterns were collected at the BL02B2 in SPring-8, Japan (λ = 0.419432 Å). The collected X-ray diffraction data were analyzed using RIETAN-FP[10] with $Bi_2YO_4X$ (X = Cl, I) ($P4/mmm$) structure model.[11] The ionization energy was measured by photoelectron yield spectroscopy (PYS; BIP-KV201, Bunkoukeiki) in a vacuum (< $5 \times 10^{-2}$ Pa).

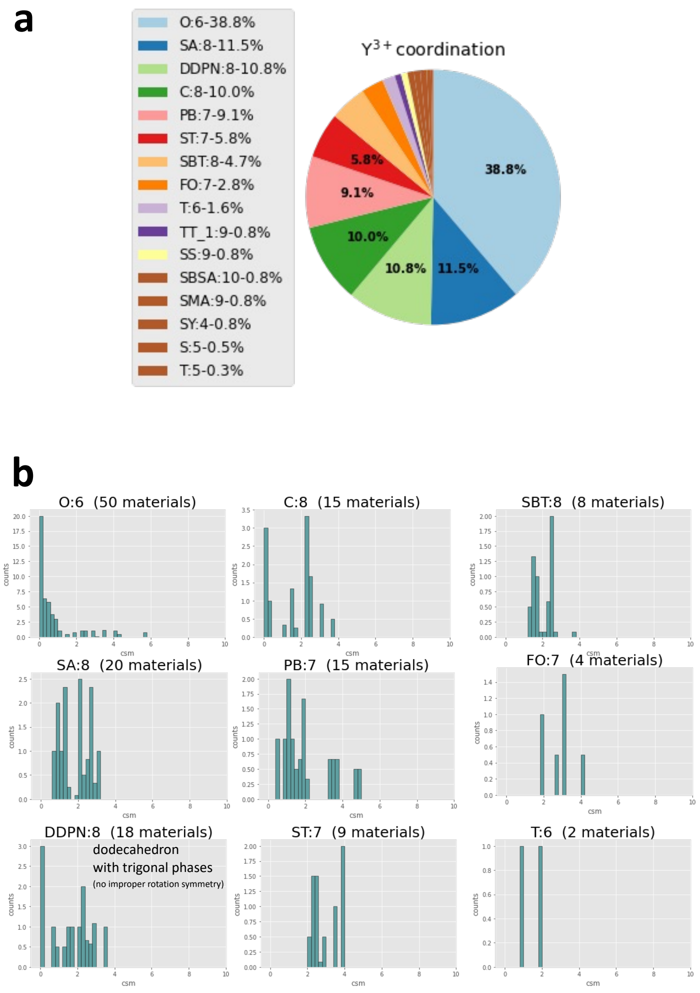


**Fig. S1.** (a) The coordination environment of $Y^{3+}$ compounds including oxygen. (b) Histogram of the CSM value of each coordination environment. The meaning of each symbol is described in the appendix.

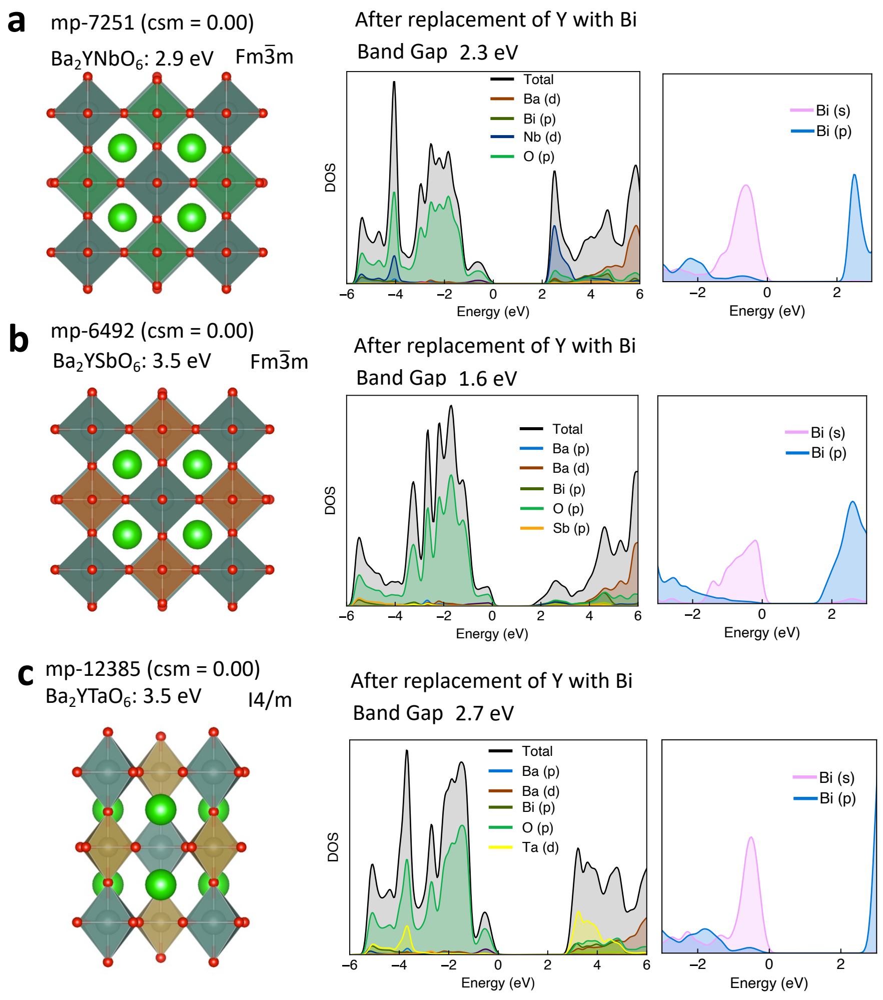


**Fig. S2.** Crystal structure and PDOS after replacing Y with Bi in (a) $Ba_2YNbO_6$, (b) $Ba_2YSbO_6$, (c) $Ba_2YTaO_6$, which have high symmetric coordination environment (CSM = 0.00) of $Y^{3+}$.

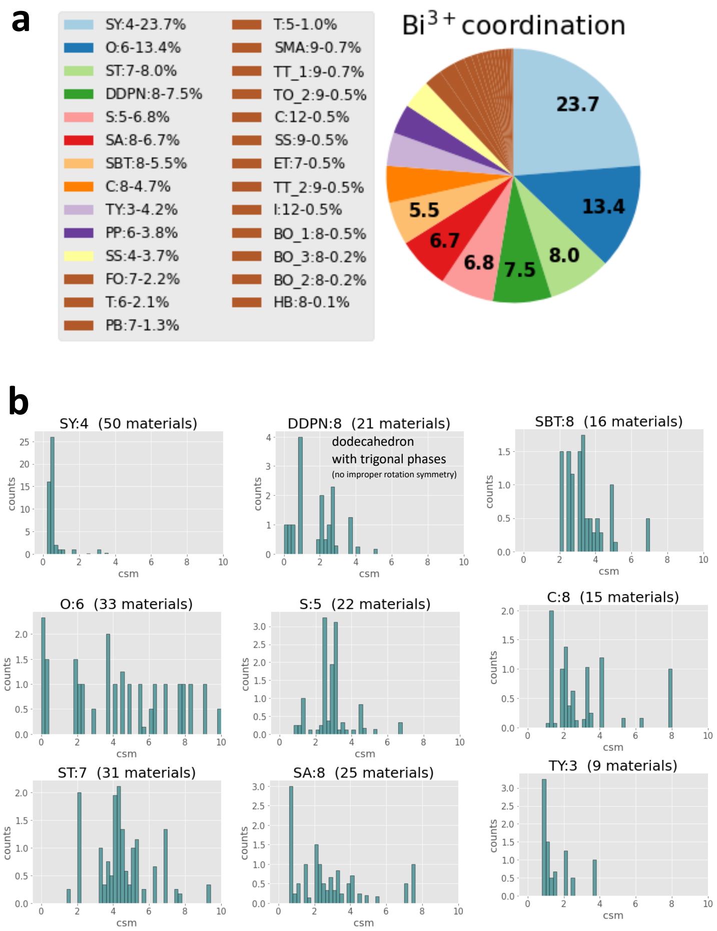


**Fig. S3.** (a) The coordination environment of $Bi^{3+}$ compounds including oxygen. (b) Histogram of the CSM value of each coordination environment. The meaning of each symbol is described in the appendix.

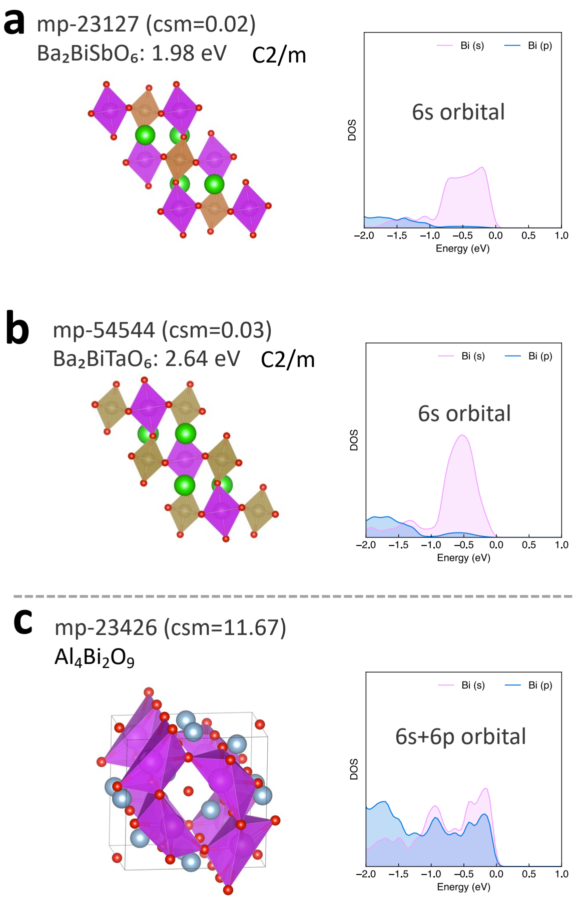


**Fig. S4.** Crystal structure and partial electronic DOS of (a) $Ba_2BiSbO_6$, (b) $Ba_2BiTaO_6$, (c) $Al_4Bi_2O_9$.

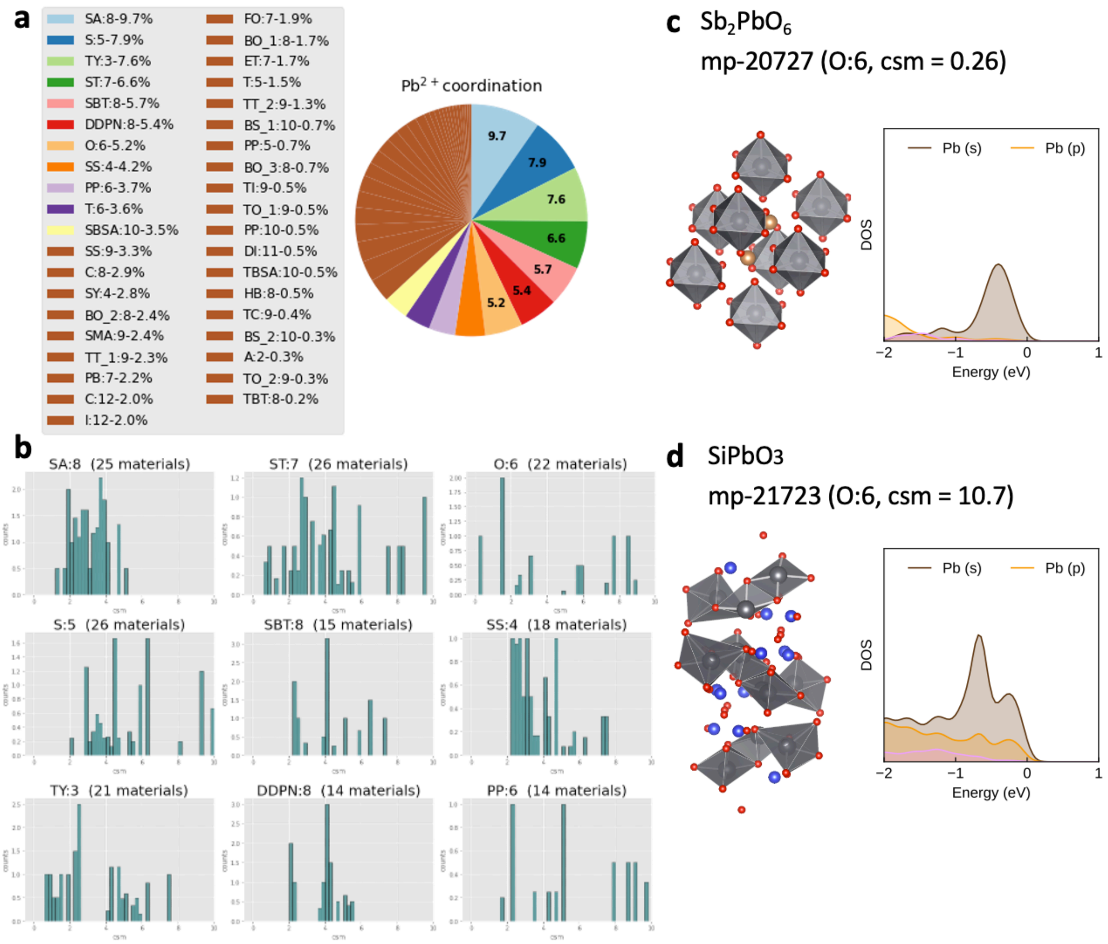


**Fig. S5.** (a) The coordination environment of $Pb^{2+}$ compounds including oxygen. (b) Histogram of the CSM value of each coordination environment. The meaning of each symbol is described in the appendix. Crystal structure and PDOS of (c) $Sb_2PbO_6$, (d) $SiPbO_3$. The highly symmetric coordination environment provides a stronger Pb 6s contribution to the VBM.

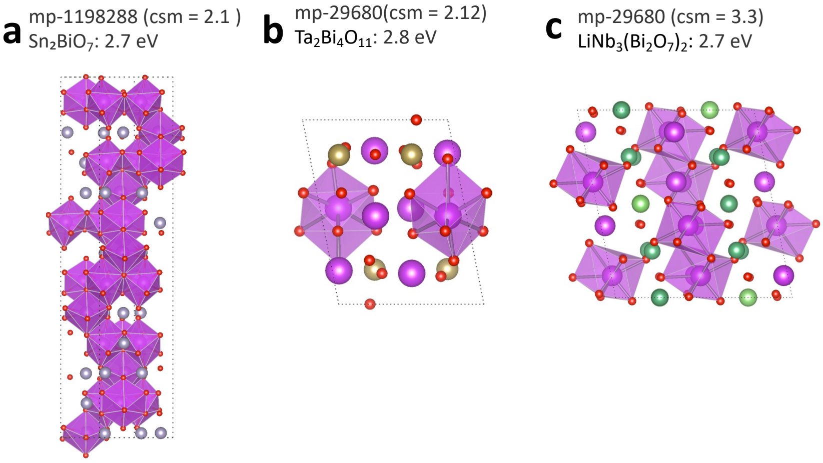


**Fig. S6.** (a-c) Crystal structure of Bi-based compounds with the smallest CSM value in C:8 environment.

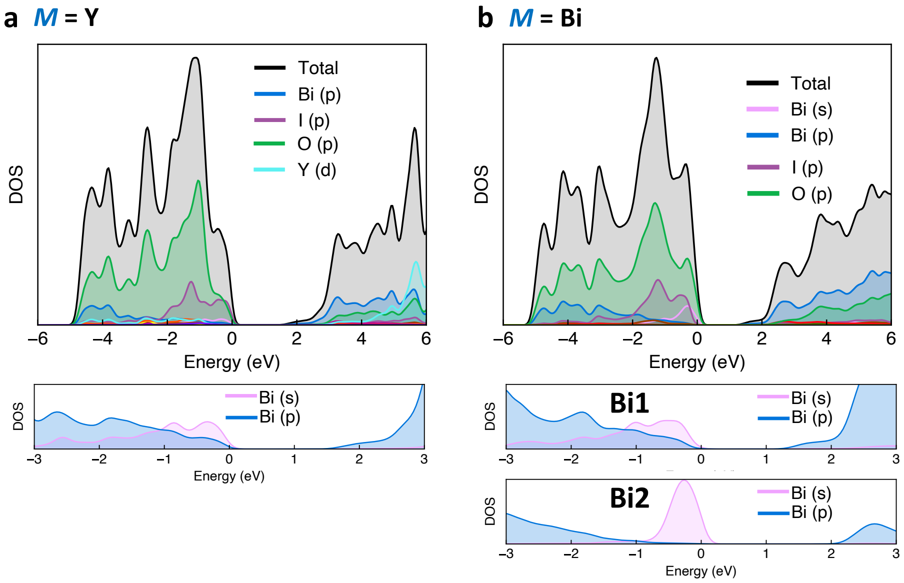


**Fig. S7.** PDOS of $Bi_2MO_4I$ with $M$ = (a) Y and (b) $M$ = Bi. The orbital contribution of each Bi is also shown.

$Bi_2MO_4X$

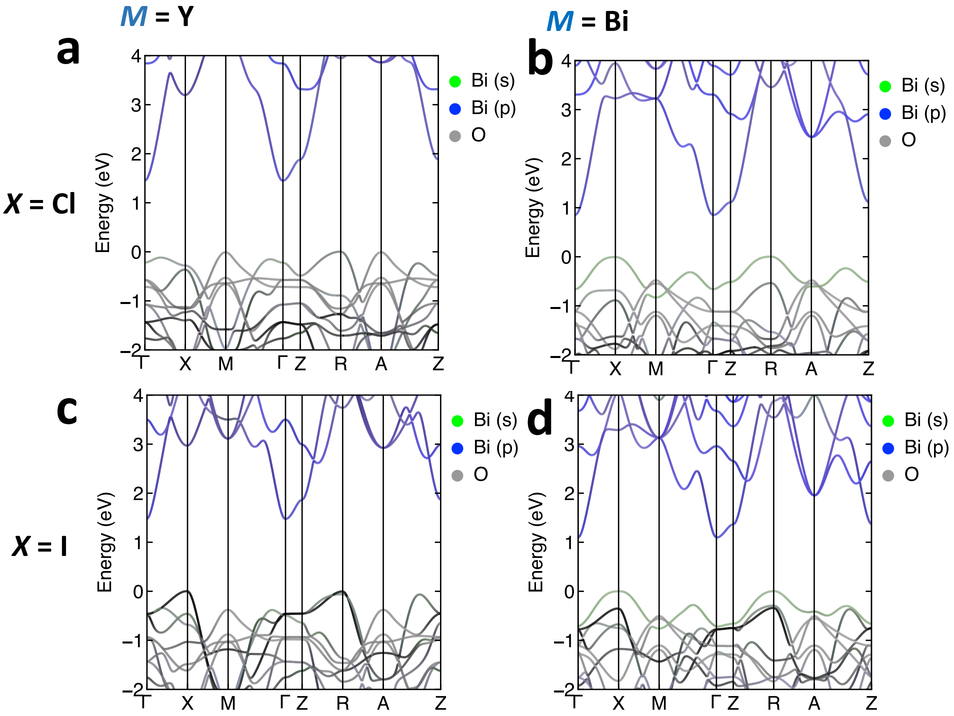


**Fig. S8.** Electronic band structure of $Bi_2MO_4X$: (a) $M$ = Y, X = Cl; (b) $M$ = Bi, X = Cl; (c) $M$ = Y, X = I; (d) $M$ = Bi, X = I.

**Table S1.** Calculated carrier effective mass of $Bi_2MO_4X$ (*M* = Y, Bi; X = Cl, I).

**a** ***M*** **= Y**

| | | $x$ | $z$ |
|---|---|---|---|
| $X$= Cl | $m_e^*/m_0$ | 0.19 | 0.43 |
| | $m_h^*/m_0$ | 0.83 | 3.03 |
| I | $m_e^*/m_0$ | 0.13 | 0.50 |
| | $m_h^*/m_0$ | 0.56 | 2.38 |

**b** ***M*** **= Bi**

| | | $x$ | $z$ |
|---|---|---|---|
| $X$= Cl | $m_e^*/m_0$ | 0.20 | 0.82 |
| | $m_h^*/m_0$ | 2.17 | 205.25 |
| I | $m_e^*/m_0$ | 0.13 | 0.75 |
| | $m_h^*/m_0$ | 1.87 | 73.56 |

**Table S2.** Born effective charges of $Bi_2MO_4X$ (*M* = Y, Bi; X = Cl, I) calculated from density functional perturbation theory.

**a** ***M*** **= Y,** ***X*** **= Cl**

| | *xx* | *yy* | *zz* |
|---|---|---|---|
| Bi1 | 5.0 | 5.0 | 2.6 |
| Y | 3.7 | 3.7 | 5.1 |
| O1 | -2.1 | -3.7 | -2.1 |
| O2 | -3.7 | -2.1 | -2.1 |
| Cl | -1.9 | -1.9 | -1.8 |

O1 (0.5, 0, 0.15), O2 (0, 0.5, 0.15)

**b** ***M*** **= Bi,** ***X*** **= Cl**

| | *xx* | *yy* | *zz* |
|---|---|---|---|
| Bi1 | 5.0 | 5.0 | 2.9 |
| Bi2 | 4.6 | 4.6 | 5.3 |
| O1 | -2.6 | -3.7 | -2.3 |
| O2 | -3.7 | -2.6 | -2.3 |
| Cl | -2.0 | -2.0 | -2.0 |

O1 (0.5, 0, 0.15), O2 (0, 0.5, 0.15)

**c** ***M*** **= Y,** ***X*** **= I**

| | *xx* | *yy* | *zz* |
|---|---|---|---|
| Bi1 | 5.3 | 5.3 | 2.9 |
| Y | 3.7 | 3.7 | 5.8 |
| O1 | -2.2 | -4.0 | -2.4 |
| O2 | -4.0 | -2.2 | -2.4 |
| I | -1.9 | -1.9 | -1.9 |

O1 (0.5, 0, 0.15), O2 (0, 0.5, 0.15)

**d** ***M*** **= Bi,** ***X*** **= I**

| | *xx* | *yy* | *zz* |
|---|---|---|---|
| Bi1 | 5.4 | 5.4 | 3.2 |
| Bi2 | 4.7 | 4.7 | 5.8 |
| O1 | -2.7 | -4.0 | -2.6 |
| O2 | -4.0 | -2.7 | -2.6 |
| I | -2.0 | -2.0 | -2.0 |

O1 (0.5, 0, 0.15), O2 (0, 0.5, 0.15)

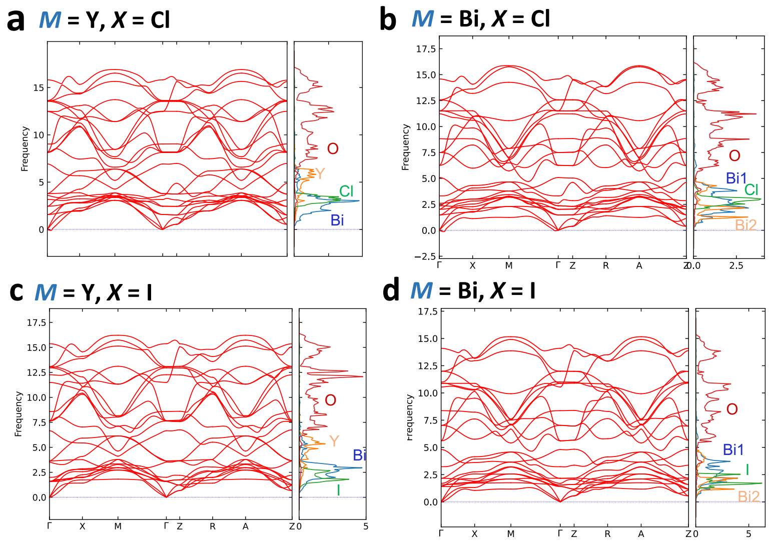


**Fig. S9.** Simulated harmonic phonon dispersions of $Bi_2MO_4X$ ( (a) $M$ = Y, X = Cl; (b) $M$ = Bi, X = Cl; (c) $M$ = Y, X = I; (d) $M$ = Bi, X = I) alongside vertical plots of the atom-projected phonon density of states.

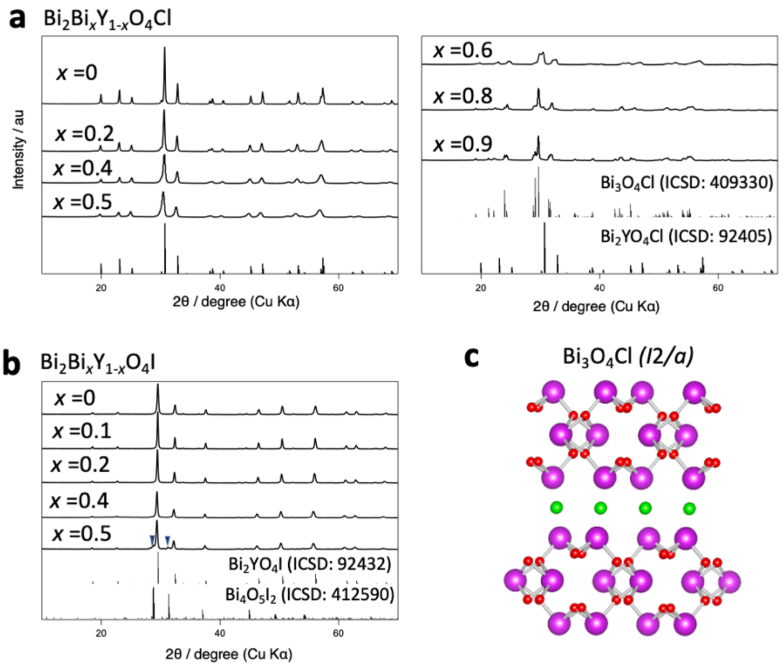


**Fig. S10.** (a, b) Measured XRD patterns of $Bi_2Bi_xY_{1-x}O_4X$ (X = Cl, I). (c) Crystal structure of the impurity phase $Bi_3O_4Cl$ ($I2/a$).

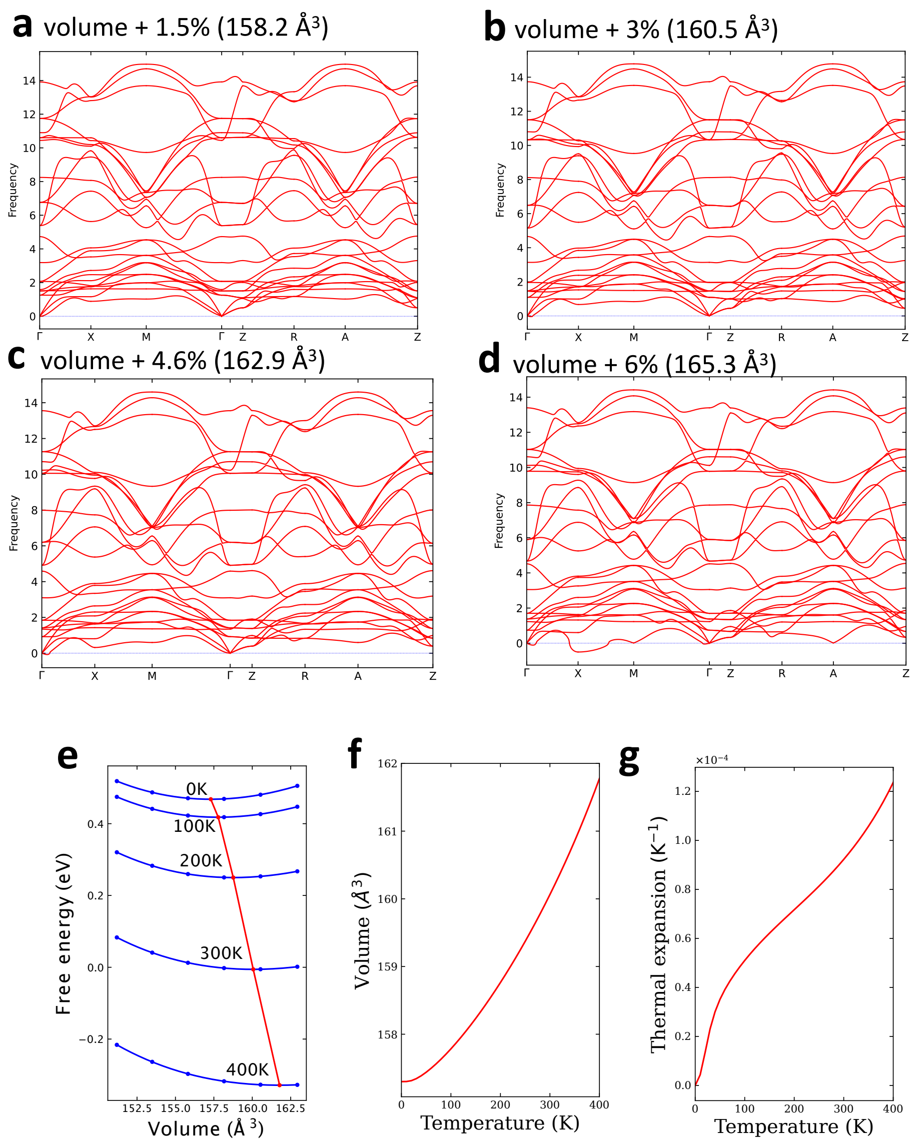


**Fig. S11.** (a–d) Simulated harmonic phonon dispersions of $Bi_2BiO_4I$ with increased volume. (e) Free energies of $Bi_2BiO_4I$ with respect to volume at temperatures from 0 to 400 K with 100 K steps are depicted by filled circles and the values are fit by the solid curves. The red line connects the energy minima of the respective curves. (g) Volumetric thermal expansion and (g) its coefficient of $Bi_2BiO_4I$. $Bi_2BiO_4I$ (*P*4/*mmm*) is dynamically stable at a low temperature. However, a dynamic instability emerges for expanded volumes at high temperatures above 400°C (d), where the produced imaginary mode is related to the movement of the symmetric Bi2. From these results, the $Bi^{3+}$ at the symmetric site is not dynamically stable at a high temperature, which results in the formation of the impurity phase having a distorted Bi site during the synthesis (Fig. S10c).

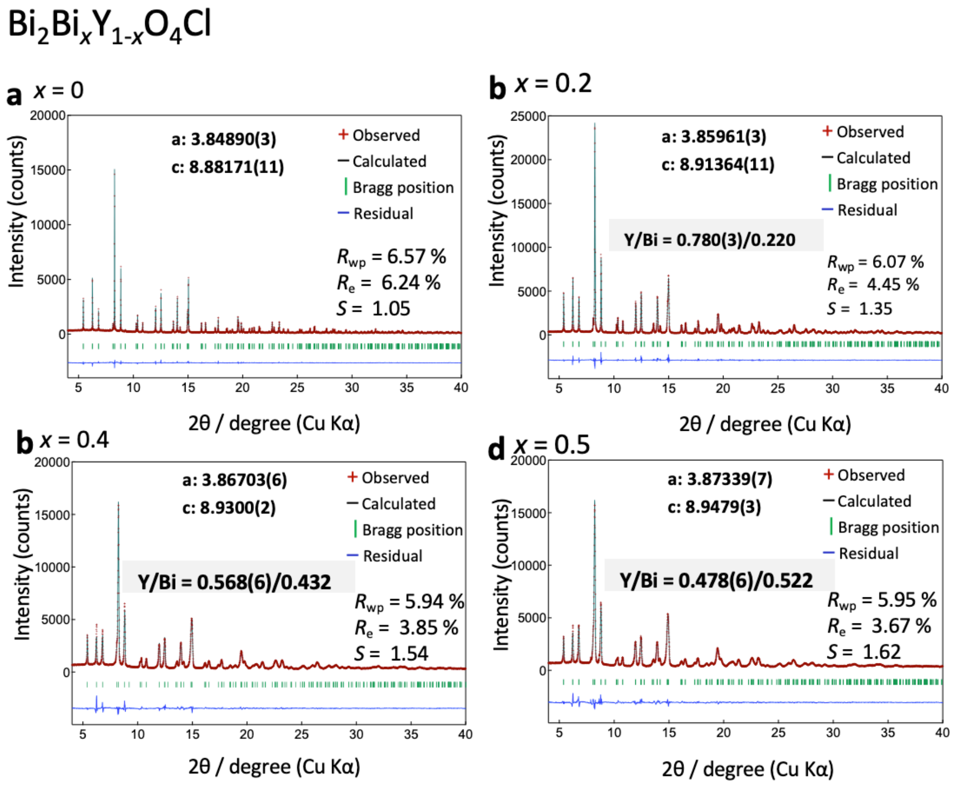


**Fig. S12.** SXRD patterns and the Rietveld refinement profiles of $Bi_2Bi_xY_{1-x}O_4Cl$ (x = (a) 0, (b) 0.2, (c) 0.4, (d) 0.5) with $Bi_2YO_4Cl$ (*P*4/*mmm*) structure model.

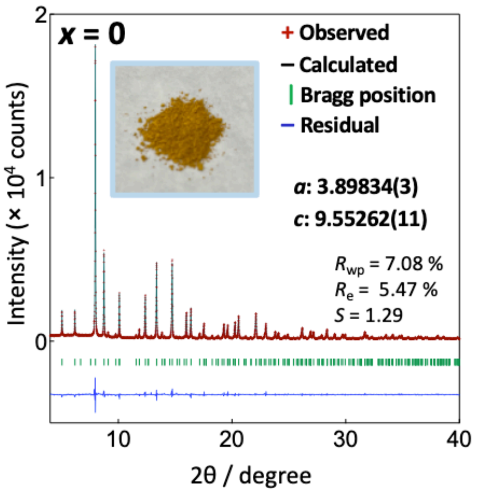


**Fig. S13.** SXRD pattern and the Rietveld refinement profile of $Bi_2Bi_xY_{1-x}O_4I$ (x = 0) with $Bi_2YO_4I$ (*P*4/*mmm*) structure model.

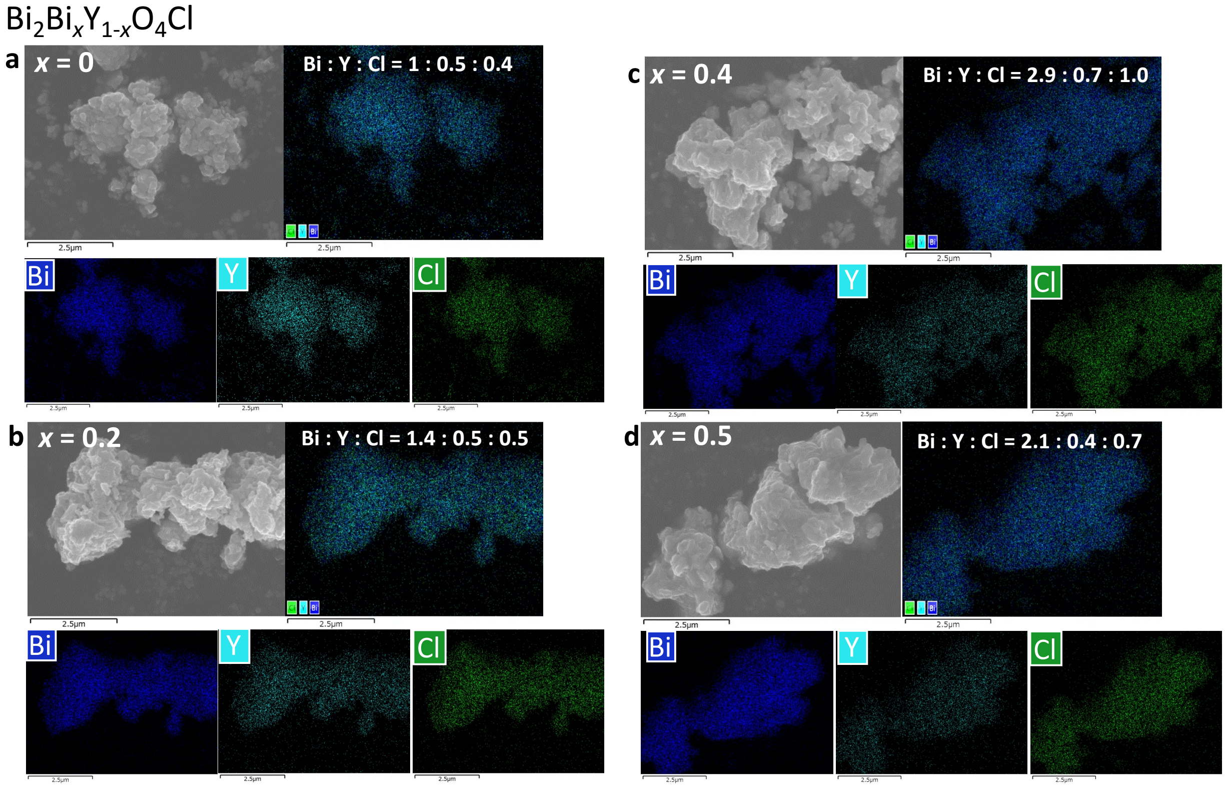


**Fig. S14.** SEM images and SEM-EDS elemental mapping of $Bi_2Bi_xY_{1-x}O_4Cl$ ($x$ = (a) 0, (b) 0.2, (c) 0.4, (d) 0.5) along with elemental molar ratios determined SEM-EDS analysis.

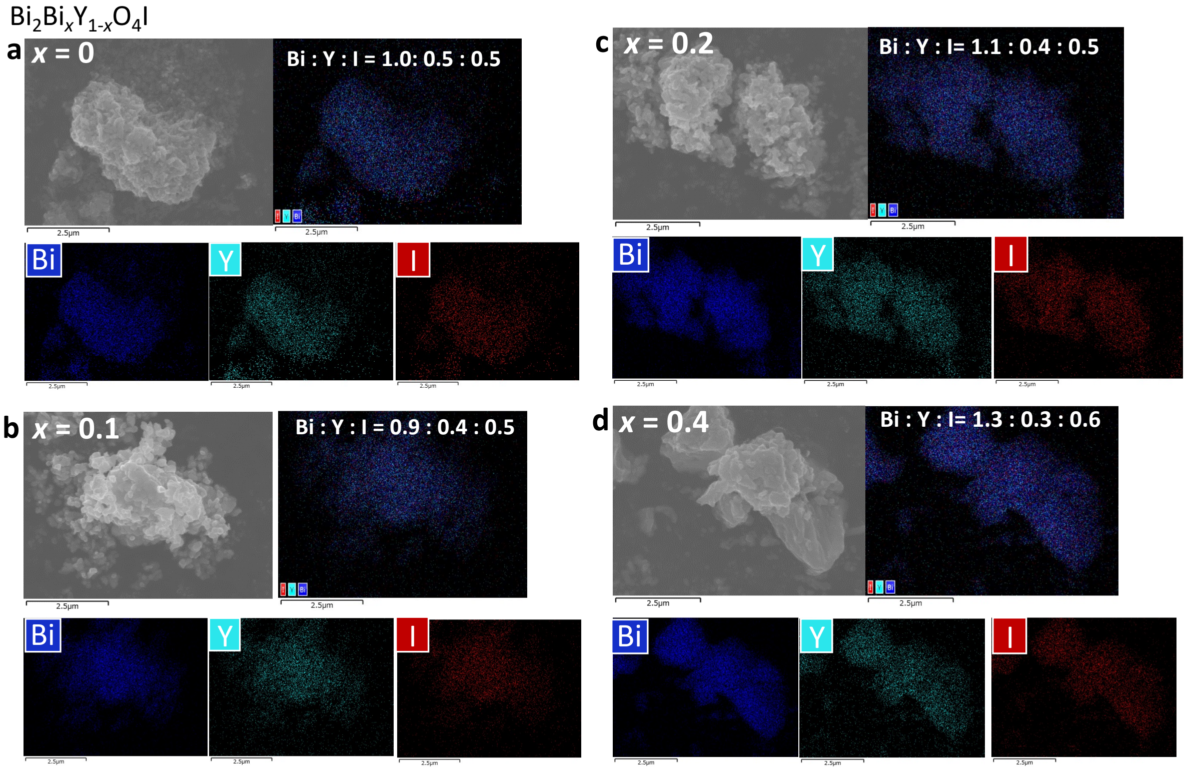


**Fig. S15.** SEM images and SEM-EDS elemental mapping of $Bi_2Bi_xY_{1-x}O_4I$ (x = (a) 0, (b) 0.1, (c) 0.2, (d) 0.4) along with elemental molar ratios determined SEM-EDS analysis.

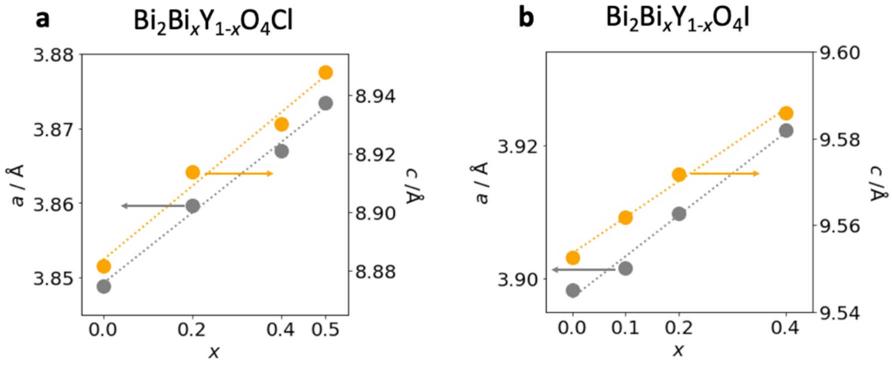


**Fig. S16.** Lattice parameters of (a) $Bi_2Bi_xY_{1-x}O_4Cl$ and (b) $Bi_2Bi_xY_{1-x}O_4I$.

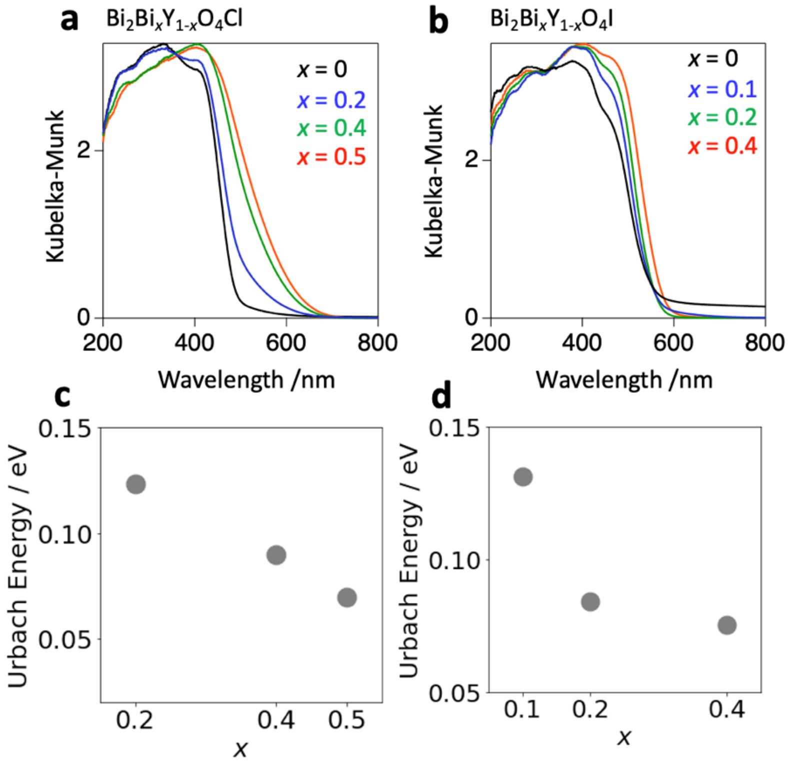


**Fig. S17.** (a, b) Diffuse reflectance spectra of $Bi_2Bi_xY_{1-x}O_4X$ (X = (a) Cl, (b) I). (c, d) The Urbach energies ($E_U$) estimated with the following equation, $\alpha(h\nu) = \alpha_0 \exp((h\nu - E_1)/E_U)$, where $\alpha_0$ and $E_1$ are fitting parameters and $E_U$ is the Urbach energy.[12,13] The $E_U$ can be estimated from the slope of ln(α) vs hν, where $d\ln(\alpha)/d(h\nu) = 1/E_U$. The Urbach Energy quantifies the broadness of the onset of absorption near the band edge, where a higher Urbach Energy represents a broader density of state.

Judging from the absence of the additional peak in the absorption spectra, the density of the state formed by Bi introduced on the Y site overlaps with the original VBM of $Bi_2YO_4X$. As the Bi introduced on the Y site increases, the density of state of the extended VBM increases, which is supported by the decreased Urbach energy with the increase of the introduced Bi.

**Appendix:** List of coordination environments

| Coordination Number | Symbol | Coordination environment |
|---|---|---|
| 4 | SY:4 | Square non-coplanar |
| 5 | S:5<br>T:5 | Square pyramid<br>Trigonal bipyramid |
| 6 | O:6<br>T:6 | Octahedron<br>Trigonal prism |
| 7 | PB:7<br>ST:7<br>ET:7<br>FO:7 | Pentagonal bipyramid<br>Square-face capped trigonal prism<br>End-trigonal-face capped trigonal prism<br>Face-capped octahedron |
| 8 | C:8<br>SA:8<br>SBT:8<br>DDPN:8<br>HB:8<br>BO 1:8<br>BO 2:8<br>BO 3:8 | Cube<br>Square antiprism<br>Square-face bicapped trigonal prism<br>Dodecahedron with triangular faces - p2345 plane normalized<br>Hexagonal bipyramid<br>Bicapped octahedron (opposed cap faces)<br>Bicapped octahedron (cap faces with one atom in common)<br>Bicapped octahedron (cap faces with one edge in common) |
| 9 | TT1:9<br>TT2:9<br>SMA:9<br>SS:9<br>TO 2:9 | Tricapped triangular prism (two square-face caps and one triangular-face cap)<br>Tricapped triangular prism (one square-face cap and two triangular-face caps)<br>Square-face monocapped antiprism<br>Square-face capped square prism<br>Tricapped octahedron (cap faces are aligned) |
| 10 | SBSA:10 | Square-face bicapped square antiprism |
| 12 | I:12<br>C:12 | Icosahedron<br>Cuboctahedron |

## Supporting References


(1) Jain, A.; Ong, S. P.; Hautier, G.; Chen, W.; Richards, W. D.; Dacek, S.; Cholia, S.; Gunter, D.; Skinner, D.; Ceder, G.; Persson, K. A. Commentary: The Materials Project: A Materials Genome Approach to Accelerating Materials Innovation. *APL Materials* **2013**, *1*, 011002.

(2) Waroquiers, D.; Gonze, X.; Rignanese, G.-M.; Welker-Nieuwoudt, C.; Rosowski, F.; Göbel, M.; Schenk, S.; Degelmann, P.; André, R.; Glaum, R.; Hautier, G. Statistical Analysis of Coordination Environments in Oxides. *Chem. Mater.* **2017**, *29*, 8346–8360.

(3) Ong, S. P.; Richards, W. D.; Jain, A.; Hautier, G.; Kocher, M.; Cholia, S.; Gunter, D.; Chevrier, V. L.; Persson, K. A.; Ceder, G. Python Materials Genomics (Pymatgen): A Robust, Open-Source Python Library for Materials Analysis. *Computational Materials Science* **2013**, *68*, 314–319.

(4) Kresse, G.; Furthmüller, J. Efficient Iterative Schemes for *Ab Initio* Total-Energy Calculations Using a Plane-Wave Basis Set. *Phys. Rev. B* **1996**, *54*, 11169–11186.

(5) Ganose, A. M.; Park, J.; Faghaninia, A.; Woods-Robinson, R.; Persson, K. A.; Jain, A. Efficient Calculation of Carrier Scattering Rates from First Principles. *Nat Commun* **2021**, *12*, 2222.

(6) M Ganose, A.; J Jackson, A.; O Scanlon, D. Sumo: Command-Line Tools for Plotting and Analysis of Periodic Ab Initio Calculations. *JOSS* **2018**, *3*, 717.

(7) Togo, A. First-Principles Phonon Calculations with Phonopy and Phono3py. *J. Phys. Soc. Jpn.* **2023**, *92*, 012001.

(8) Togo, A.; Chaput, L.; Tanaka, I.; Hug, G. First-Principles Phonon Calculations of Thermal Expansion in $Ti_3SiC_2$, $Ti_3AlC_2$, and $Ti_3GeC_2$. *Phys. Rev. B* **2010**, *81*, 174301.

(9) Shannon, R. D.; Waring, R. K. Synthesis and Characterization of a New Series of $BiOI_{1-x-y}Br_xCl_y$, Pigment. *J. Phys. Chem. Solids.* **1985**, *46*, 325–330.

(10) Izumi, F.; Momma, K. Three-Dimensional Visualization in Powder Diffraction. *Solid State Phenom.* **2007**, *130*, 15–20.

(11) Schmidt, M.; Oppermann, H.; Hennig, C. Untersuchungen zu Bismutseltenerdoxidhalogeniden der Zusammensetzung $Bi_2SEO_4X$ (X = Cl, Br, I). *Z. Anorg. Allg. Chem.* **2000**, 11.

(12) Urbach, F. The Long-Wavelength Edge of Photographic Sensitivity and of the Electronic Absorption of Solids. *Phys. Rev.* **1953**, *92*, 1324–1324.

(13) Pankove, J. I. Absorption Edge of Impure Gallium Arsenide. *Phys. Rev.* **1965**, *140*, A2059–A2065.
